# Combined Transcriptome and Proteome Profiling for Role of pfEMP1 in Antimalarial Mechanism of Action of Dihydroartemisinin

**DOI:** 10.1128/Spectrum.01278-21

**Published:** 2021-12-15

**Authors:** Lina Chen, Zhongyuan Zheng, Hui Liu, Xi Wang, Shuiqing Qu, Yuanmin Yang, Shuoqiu Deng, Yu Zhang, Liu Tuo, Yongdan Zhao, Yujie Li

**Affiliations:** a Artemisinin Research Center, Institute of Chinese Materia Medica, China Academy of Chinese Medical Sciences, Beijing, China; b School of Pharmacy, Shanxi Medical University, Taiyuan, Shanxi, China; University of Georgia

**Keywords:** dihydroartemisinin, membrane proteome, pfEMP1, transcriptome

## Abstract

Malaria parasites induce morphological and biochemical changes in the membranes of parasite-infected red blood cells (iRBCs) for propagation. Artemisinin combination therapies are the first-line antiplasmodials in countries of endemicity. However, the mechanism of action of artemisinin is unclear, and drug resistance decreases long-term efficacy. To understand whether artemisinin targets or interacts with iRBC membrane proteins, this study investigated the molecular changes caused by dihydroartemisinin (DHA), an artemisinin derivative, in Plasmodium falciparum 3D7 using a combined transcriptomic and membrane proteomic profiling approach. Optical microscopy and scanning electron microscopy showed that DHA can cause morphological variation in the iRBC membrane. We identified 125 differentially expressed membrane proteins, and functional analysis indicated structural molecule activity and protein export as key biological functions of the two omics studies. DHA treatment decreased the expression of *var* gene variants *PF3D7_0415700* and *PF3D7_0900100* dose-dependently. Western blotting and immunofluorescence analysis showed that DHA treatment downregulates the *var* gene encoding P. falciparum erythrocyte membrane protein-1 (pfEMP1). pfEMP1 knockout significantly increased artemisinin sensitivity. Results showed that pfEMP1 might be involved in the antimalarial mechanism of action of DHA and pfEMP1 or its regulated factors may be further exploited in antiparasitic drug design. The findings are beneficial for elucidating the potential effects of DHA on iRBC membrane proteins and developing new drugs targeting iRBC membrane.

**IMPORTANCE** Malaria parasites induce morphological and biochemical changes in the membranes of parasite-infected red blood cells (iRBCs) for propagation, with artemisinin combination therapies as the first-line treatments. To understand whether artemisinin targets or interacts with iRBC membrane proteins, this study investigated the molecular changes caused by dihydroartemisinin (DHA), an artemisinin derivative, in Plasmodium falciparum 3D7 using a combined transcriptomic and membrane proteomic profiling approach. We found that DHA can cause morphological changes of iRBC membrane. Structural molecule activity and protein export are considered to be the key biological functions based on the two omics studies. pfEMP1 might be involved in the DHA mechanism of action. pfEMP1 or its regulated factors may be further exploited in antiparasitic drug design. The findings are beneficial for elucidating the potential effects of DHA on iRBC membrane proteins and developing new antimalarial drugs targeting iRBC membrane.

## INTRODUCTION

Malaria, caused by *Plasmodium* spp., including P. falciparum, P. vivax, P. ovale, P. malariae, and P. knowlesi, is a major public health challenge and was prevalent in 91 countries in 2019, with 229 million new cases reported and nearly 409,000 deaths (1), indicating that it is still one of the deadliest infectious diseases affecting humans. Artemisinin-based combination therapies (ACTs) are recommended as first-line treatments for malaria and have saved the lives of millions of patients who do not achieve the treatment goal with adequate chloroquine, quinine, chloroquine, mefloquine, and sulfonamide. However, drug resistance poses an eminent danger to malaria elimination efforts worldwide. Delayed parasite clearance by ACT in clinical trials has been reported in Cambodia (2–6). This region is historically described as a site of emerging resistance to earlier antimalarial first-line therapies, which later rapidly spread across Africa. In recent years, resistance to artemisinin has also been found in Africa (7), where malaria transmission is consistently high (8). Thus, clarification of the mechanism of action of artemisinin could promote the development of better strategies to cure malaria in the era of artemisinin resistance. Avoiding resistance is necessary to improve clinical outcomes. Understanding the biological functions of apicomplexan rhomboids is an active area of research because of the critical roles identified for several of these molecules in host cell invasion and pathogenesis. The antimalarial activity of artemisinin is an active area of research. Artemisinin exerts its antimalarial effect by binding to cytosolic and mitochondrial targets through its unique endoperoxide bridge structure in order to interfere with the signaling pathway or disturb electron transport in a peroxide-dependent manner (9). In addition, parasite proteins PfTCTP, PfATP6, PfPI3K, PfCRT, and PfMDR1 bind to artemisinin, directly killing *Plasmodium* (10). Artemisinin, as a prodrug, is considered to be activated specifically by heme (11), ferrous ions (12), or *Plasmodium* mitochondria (13), which all seem essential for its antimalarial activity.

According to Wang et al., heme, rather than ferrous ions or *Plasmodium* mitochondria, is an artemisinin-specific activator (11). In fact, *Plasmodium* consumes up to 80% of host hemoglobin and releases monomeric heme, which is toxic and insoluble in water and can interact with heme-binding proteins, lipoproteins, and cell membranes (14). Most importantly, substantial amounts of free heme, on average >50%, escape polymerization during the entire parasite cycle (15). Free heme in food vacuoles can flow into the parasite cytosol (16), entering the red blood cell (RBC) cytoplasm. Clinically, intravascular hemolysis is usually found in severe malaria patients, with ∼40% of the hemoglobin in each parasite-infected RBC (iRBC) being released and easily oxidized (17), resulting in the release of free heme and its accumulation in plasma (18–20). Free heme in plasma played a key role in the pathogenesis of a cerebral malaria mouse model by destroying the blood-brain barrier (21). Given the presence of free heme both inside and outside iRBCs, we hypothesize that artemisinin can be activated inside and outside iRBCs and attacks nearby molecules or structures, such as the iRBC membrane (18). Therefore, from the host cell point of view, it is still unclear which iRBC membrane proteins will be affected by free radicals produced by artemisinin, so studies on the membrane proteins of artemisinin-treated iRBCs are important. Several studies have reported the effects of antimalarial drugs on the iRBC membrane. Zhang et al. reported that chloroquine and artesunate do not directly influence the shear modulus of iRBCs (22). Byeon et al. investigated the effects of chloroquine on the mechanical property variation of iRBCs (23). However, these studies did not focus on iRBC membrane proteins after treatment with dihydroartemisinin (DHA), an artemisinin derivative.

This study used a combined transcriptomic and membrane proteomic profiling approach to obtain systematic insight into the molecular changes in the iRBC membrane after DHA treatment of P. falciparum 3D7 at ring and trophozoite stages. Critical differentially expressed genes (DEGs) and proteins were estimated by bioinformatics analysis. Validation by quantitative reverse transcription PCR (qRT-PCR) and Western blotting was performed to identify the iRBC membrane hub proteins. Finally, we investigated whether P. falciparum erythrocyte membrane protein-1 (pfEMP1) is involved in the antimalarial action of artemisinin using a *var* gene-knockout parasite. The findings would help in understanding the drug-related interaction with iRBCs at the molecular level and developing new drugs targeting the iRBC membrane.

## RESULTS

### DHA effects on the morphology and protein of iRBC membranes.

**FIG 1:**
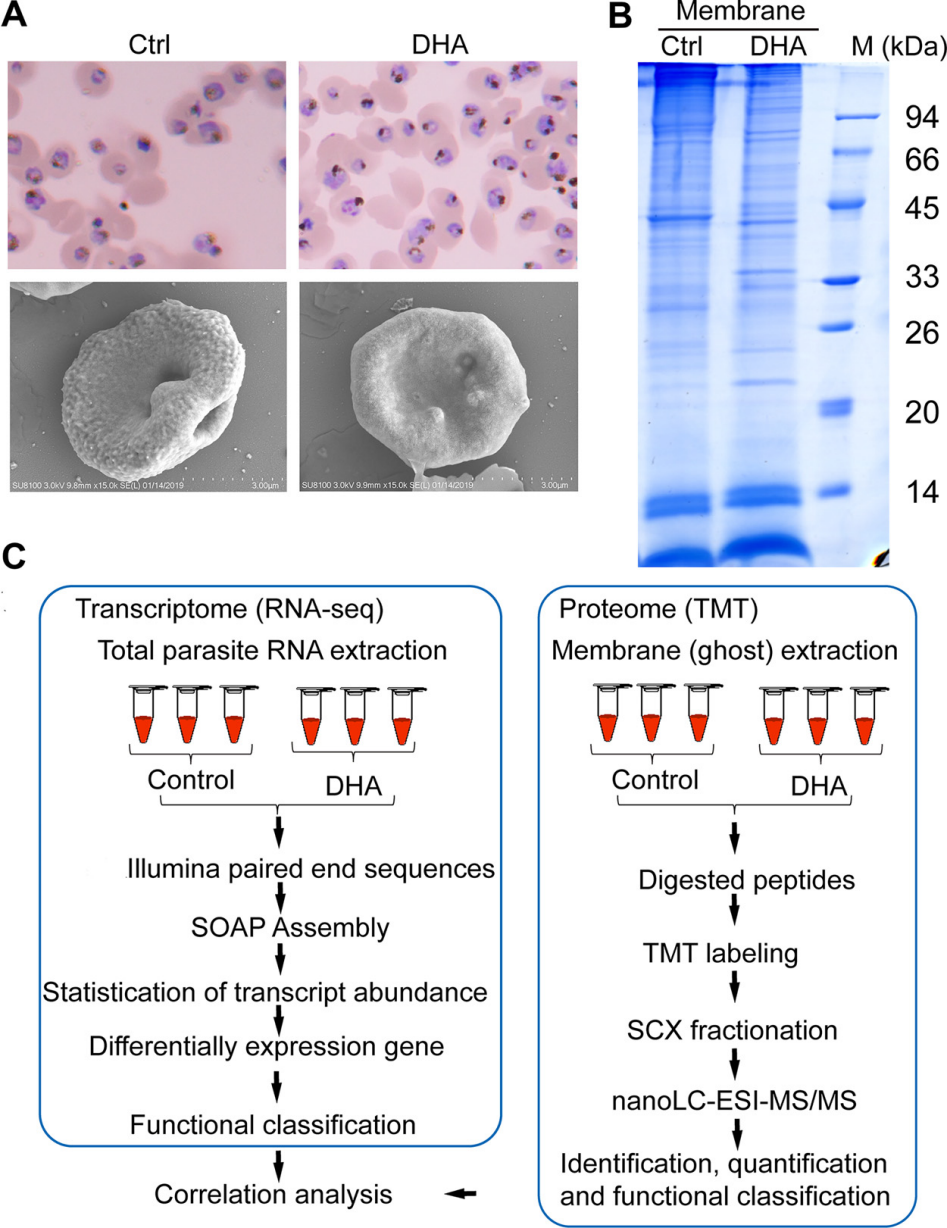
Effect of dihydroartemisinin (DHA) on the morphology and protein of the parasite-infected red blood cells (iRBC) membrane. (A) Giemsa staining (upper panel) and scanning electron microscopy (SEM) images (lower panel) of Plasmodium falciparum 3D7 in control and DHA groups. Ctrl, control. (B) One-dimensional electrophoresisIs (DE) coomassie-stained gel of iRBC membrane proteins. Ctrl, control; M, marker. (C) Flow diagram of the omics analysis process.

We used DHA to treat synchronized P. falciparum 3D7 in the early stage. Microscopic images (Fig. 1A, upper panel) show that after 500 nM DHA treatment (invading the trophozoite period of 24 to 30 h) for 4 h, the DHA group showed shrinkage and cytoplasm deformation, while the control group was healthy. Scanning electron microscopy (SEM) showed a raised knob-like structure on the iRBC membrane of the DHA group compared to the smooth surface topography of the control group. The total protein of the iRBC plasma membrane in the DHA group was downregulated compared with that in the control group (Fig. 1B), and high- and low-molecular-weight proteins were distributed. These results showed that DHA can affect the iRBC membrane. To further analyze the mechanism of the antimalarial action of DHA, we designed an experiment, as shown in Fig. 1C.

### Transcriptome sequencing analysis.

To investigate the gene expression changes in P. falciparum 3D7 after DHA treatment, triplicate samples from the control and DHA groups were used to construct cDNA libraries, and transcriptome sequencing was performed using BGISEQ-500 sequencing, yielding 334.3 million raw reads. The quality control of sequencing data is presented in Tables S1 and S2 and Table 1. The Q30 percentages for all clean reads in the libraries were >90%. Illumina paired-end sequencing of 6 samples yielded 308,346,652 clean reads.

**TABLE 1:** Percentage of genes expressed in ctrl1, ctrl2, ctrl3, DHA1, DHA2, and DHA3 samples^*a*^

FPKM interval	No. (%) genes expressed in:					
	ctrl1	ctrl2	ctrl3	DHA1	DHA2	DHA3
0–1	860 (14.93%)	844 (14.65%)	865 (15.01%)	831 (14.42%)	838 (14.55%)	844 (14.65%)
1–3	585 (10.15%)	604 (10.48%)	604 (10.48%)	640 (11.11%)	647 (11.23%)	657 (11.40%)
3–15	1,341 (23.28%)	1,335 (23.17%)	1,328 (23.05%)	1,398 (24.27%)	1,396 (24.23%)	1,382 (23.99%)
15–60	1,427 (24.77%)	1428 (24.79%)	1,419 (24.63%)	1,244 (21.59%)	1,245 (21.61%)	1,241 (21.54%)
>60	1548 (26.87%)	1,550 (26.91%)	1,545 (26.82%)	1,648 (28.61%)	1,635 (28.38%)	1,637 (28.42%)

a ctrl, control; DHA, dihydroartemisinin; FPKM, fragments per kilobase of transcript per million mapped reads.

Using principal-component analysis (Fig. S1), samples from the control and DHA groups, each with three biological replicates, were further divided into ctrl1, ctrl2, ctrl3, DHA1, DHA2, and DHA3 groups. Gene expression levels between replicates for each sample exhibited a high correlation coefficient, indicating good repeatability between replicates. More than 99% of genes with fragments per kilobase of transcript per million mapped reads (FPKM) values of >0 were detected in the 6 samples (Table S1), and the percentage of genes expressed in the ctrl1, ctrl2, ctrl3, DHA1, DHA2, and DHA3 samples was indicated. Based on the gene FPKM values, the expression levels of all genes were classified into four categories. After DHA treatment, the majority of genes with FPKM values of 10 < FPKM < 100 were considered moderately expressed, and ∼25% of genes with FPKM values of 0< FPKM <10 were considered to have low expression. The number of nonexpressed genes (FPKM = 0 or undetected) accounted for ∼2% of the total genes after DHA treatment.

### Identification and functional enrichment analysis of the most relevant genes.

**FIG 2:**
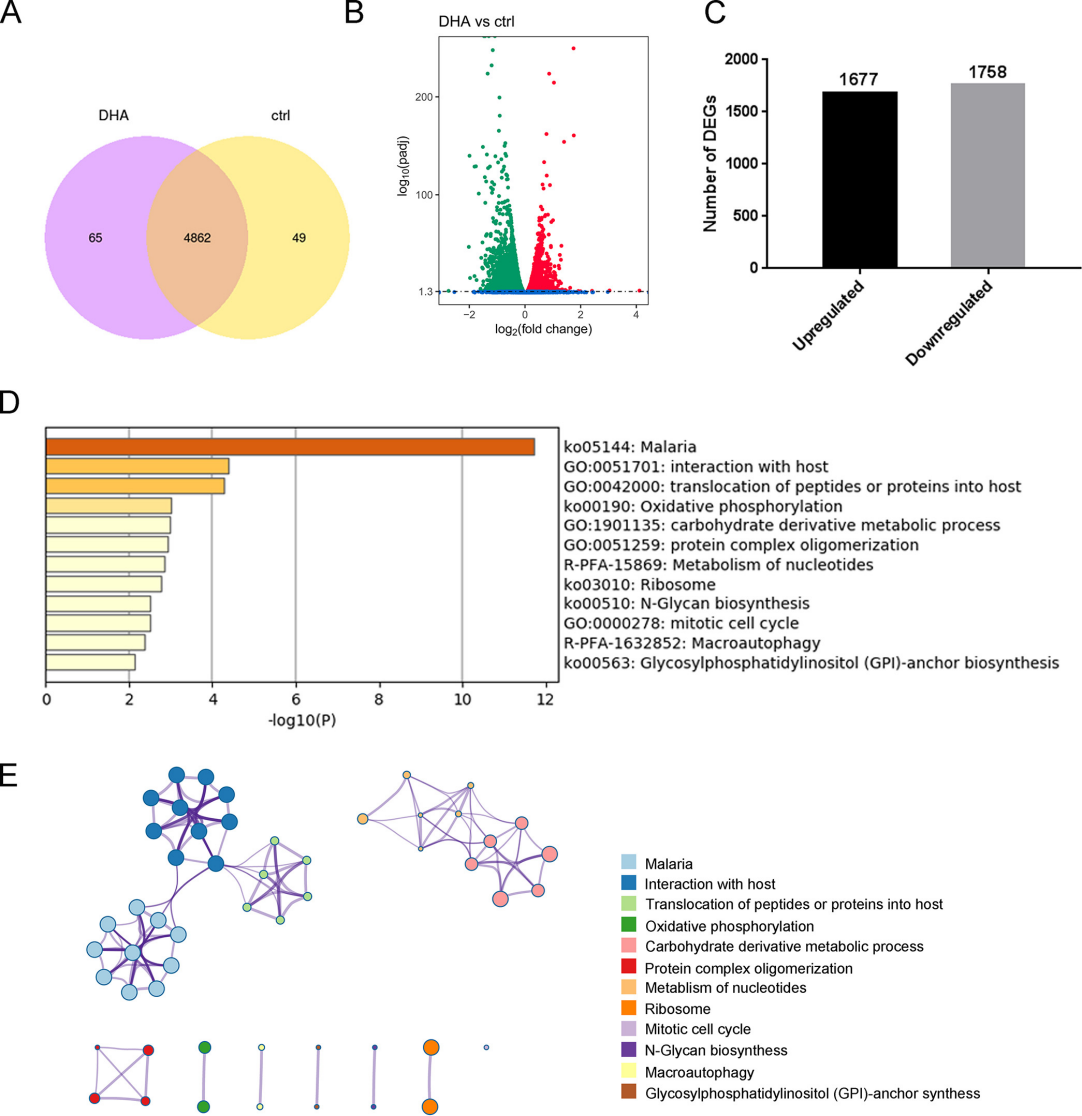
Identification and functional enrichment analysis to identify the most relevant genes after dihydroartemisinin (DHA) treatment. (A) Venn diagram showing the overlap between genes expressed in the control and DHA groups. (B) Volcano plot displaying differentially expressed genes (DEGs) in the two groups. The *y* axis shows the mean expression value of log_10_ (*q* value), and the *x* axis displays the log_2_ fold change (FC) value. Blue dots represent transcripts that did not reach statistical significance (*q *> 0.05); green dots and red dots represent transcripts whose expression levels were significantly different (green dots: down-regulated; red dots: up-regulated, *q *< 0.05). (C) Number of DEGs found between DHA and control groups. The number of DEGs is shown on the top of the histograms. (D) Heatmap of enriched terms across common DEGs compared between DHA and control groups, colored by *P* values. (E) Network of enriched terms are colored by the cluster ID, and nodes sharing the same cluster are typically close to each other. (D and E) Produced using metascape (http://metascape.org/gp/index.html#/main/step1).

We identified 4,927 and 4,911 genes in the DHA and control groups, respectively (Fig. 2A). Of these, 4,862 genes overlapped in the two samples, and 65 and 49 genes were specific to DHA and control groups, respectively (Fig. 2A). Of the 4,862 genes, 3,435 DEGs were detected significantly differently between the two groups. Of these 3,435 DEGs, 1,677 were upregulated, while 1,758 were downregulated (Fig. 2B and C and Table S3). These results suggested that the gene expression profile after DHA treatment is unique compared to that of the control group.

The top 60 genes upregulated by DHA treatment were selected to show their main functions, which included *Duffy-binding-like merozoite surface protein 2* (*dblmsp2*), *exported protein family 1* (*epf1*), *reticulocyte binding protein homolog 5* (*rh5*), sporozoite and liver-stage asparagine-rich protein, *cysteine repeat modular protein 2* (*crmp2*), *erythrocyte binding antigen-181* (*eba181*), *potassium channel k2*, *surface-associated interspersed protein 1.1* (*surf1.1*), mechanosensitive ion channel protein, and *sporozoite invasion-associated protein 1* (*siap1*) with annotations and several genes without annotations. We further analyzed transcripts that were at least 3-fold downregulated. The top 60 genes downregulated by DHA treatment included *rifin* (*rif*), *mature parasite infected erythrocyte surface antigen* (*mesa*), *1-cys peroxiredoxin* (*1-cyspxn*), *protein kinase 7* (*pk7*), P. falciparum *maurer's cleft two transmembrane protein* (*pfmc-2tm*), *msp7-like protein* (*msrp6*), and *var* with annotations and various genes without annotations (Table S3). More genes involved in membrane structure than merozoite- and sporozoite-associated genes were found to be downregulated.

DEGs were classified using pathway and process enrichment analysis to evaluate their potential functions in the DHA group. As shown by metascape, the DEGs were enriched mainly in interaction with the host (44 genes), translocation of peptides or proteins into the host (10 genes), carbohydrate derivative metabolic process (23 genes), protein complex oligomerization (4 genes; Gene Ontology [GO]), malaria, oxidative phosphorylation, ribosome, N-glycan biosynthesis, glycosylphosphatidylinositol (GPI)-anchor biosynthesis (KEGG), metabolism of nucleotides, and macroautophagy (reactome) (Fig. 2D and E and Table S4). A notable finding from this analysis was the interaction with the host item to obtain novel information about the effect of DHA treatment on parasite growth. Within the enriched KEGG pathways, we found that the malaria pathway (ko05144) was associated with 85 genes.

A comparison between transcriptome changes induced by DHA treatment and parasite morphology demonstrated that DHA has a strong and long-lasting effect on the iRBC membrane structure. DHA specifically recognized the iRBC membrane, as shown by the downregulated genes, and initiated a stress response in the parasites, as shown by the upregulated genes in the transcriptome.

### Proteomics analysis of the iRBC membrane after DHA treatment.

To detect and reveal changes in iRBC membrane proteins after DHA treatment, proteomes of the P. falciparum 3D7 samples (three biological replicates in each group) were analyzed using tandem mass tag (TMT) labeling proteomics technology. Proteome sequencing yielded 571,907 spectra, and after removing the peptides with a false-discovery rate (FDR) of >1%, we identified 6,080 peptides and 1,013 proteins. More than 56% of the proteins included at least 2 peptides, and 991 proteins were used to perform global protein expression profile analysis. We identified 15 low-molecular-weight (*M*_r_ < 10 kDa) and 4 high-molecular-weight (*M*_r_ > 560) proteins using TMT quantitative proteomics technology. Figure S2A shows the distribution of the peptides’ length and number. Most peptides had a length of 7 to 8 amino acids (aa), and the average peptide length was 10.12 aa, which was within the reasonable range of peptide lengths. The protein mass and sequence coverage of proteins (Fig. S2B) were also obtained.

To screen differentially expressed proteins (DEPs), the screening conditions were as follows: significant upregulation, fold change (FC) ≥ 1.2 and *P ≤ *0.05; significant downregulation, FC ≤ 2/3 and *P ≤ *0.05. Compared with the control group, the DHA group had 125 DEPs, of which 31 were upregulated (Table S5) and 94 were downregulated (Table S6).

Compared with the control group, among the significant DEPs, 40S ribosomal protein S2 and MSP7-LIKE PROTEIN (MSRP6) showed the highest up- and downregulation, respectively, in the DHA group. Notably, an array of downregulated *Plasmodium* proteins, such as *Plasmodium* EXPORTED PROTEIN (PHISTa), PHISTb, PHISTc, RIFIN, and STEVOR, were involved in many biological processes, such as pfEMP1 transport, cell adhesion, gametocytogenesis, cell rigidity changes, and pregnancy-related malaria. Therefore, PHIST has been reported to be the core of host cell remodeling. However, HEXOKINASE, deoxyhypusine hydroxylase, and other structural proteins were upregulated, indicating that DEPs may play an important role in regulating the iRBC membrane during DHA treatment.

**FIG 3:**
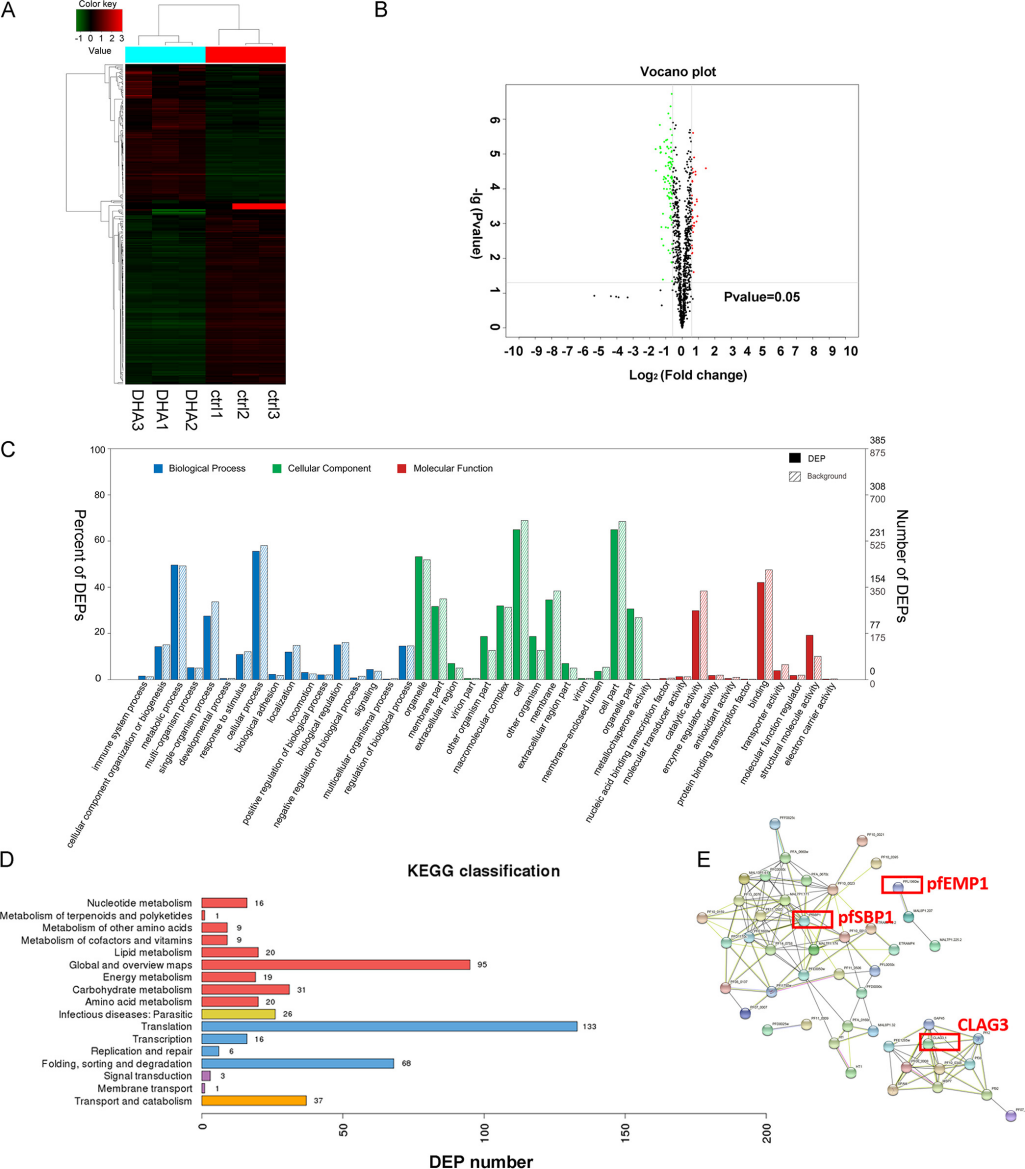
Proteomics analysis of the parasite-infected red blood cell (iRBC) membrane after dihydroartemisinin (DHA) treatment. (A) Unsupervised hierarchical cluster analysis of differentially expressed proteins (DEPs). (B) Volcano plot displaying DEPs within two groups. The *y* axis shows the mean expression value of log_10_ (*q* value), and the *x* axis displays the log_2_ fold change (FC) value. Black dots represent proteins that did not reach statistical significance (*q *> 0.05); red and green dots represent proteins whose expression levels were significantly different (*q *< 0.05). (C) Gene Ontology (GO) function classification of DEPs. The abscissa is the content of the secondary function classification of the GO database, and the ordinate is the percentage of DEPs contained in the corresponding secondary functional classification (left) and number (right). (D) Kyoto Encyclopedia of Genes and Genomes (KEGG) classification of DEPs. Number of annotated proteins (*x* axis) versus KEGG categories (*y* axis). (E) STRING analysis of proteins decreased in the ring stage of Plasmodium falciparum 3D7 after DHA treatment. Proteins are shown as nodes, and the color of each link defines the type of evidence available for the interaction between two proteins (e.g., blue: cooccurrence; black: coexpression; purple: experimental; aqua: databases; green: text mining; light blue: homology).

To further analyze DEPs, we performed unsupervised hierarchical cluster analysis. Clustering analysis of the six samples was done according to the whole identified proteins and differential proteins. The results of three independent replicates of each group are shown in Fig. 3A.

GO enrichment analysis showed that the DEGs were significantly enriched in GO terms. All the classifications listed here are based on the gene enrichment principle; that is, the frequency of these classes of proteins in our results is much higher than the frequency of occurrence of these classes in the *Plasmodium* gene (*P < *0.05) (Fig. 3B). Specifically, the metabolic process, cellular process, single-organism process, biological regulation, localization, and biological adhesion were the main categories in biological processes. The organelle, membrane part, other organism part, cell, and cell part accounted for the large items in the cellular component. Binding, catalytic activity, and structural molecule activity formed the major proportion in molecular function (Fig. 3C and Table S7). We also performed KEGG pathway analysis on iRBC membrane proteins using the Molecule Annotation System (MAS 3.0). Results indicated that significant DEPs were enriched mostly in two pathways involved in translation, transport, and catabolism (Fig. 3D and Table S8). It is likely that numerous enzymes and related proteins might play a key role in the major pathways in DHA’s mode of action. In addition, protein-protein interactions (PPIs) using STRING analysis were analyzed to determine the relationship of important proteins and find hub proteins involved in DHA pharmacology. Results showed that proteins that decreased in the ring stage of P. falciparum 3D7 after DHA treatment are associated with the cell-cell adhesion process network, including the important proteins pfEMP1, CLAG3, and pfSBP1 (Fig. 3E).

### Correlation analysis of transcriptomics and proteomics.

**FIG 4:**
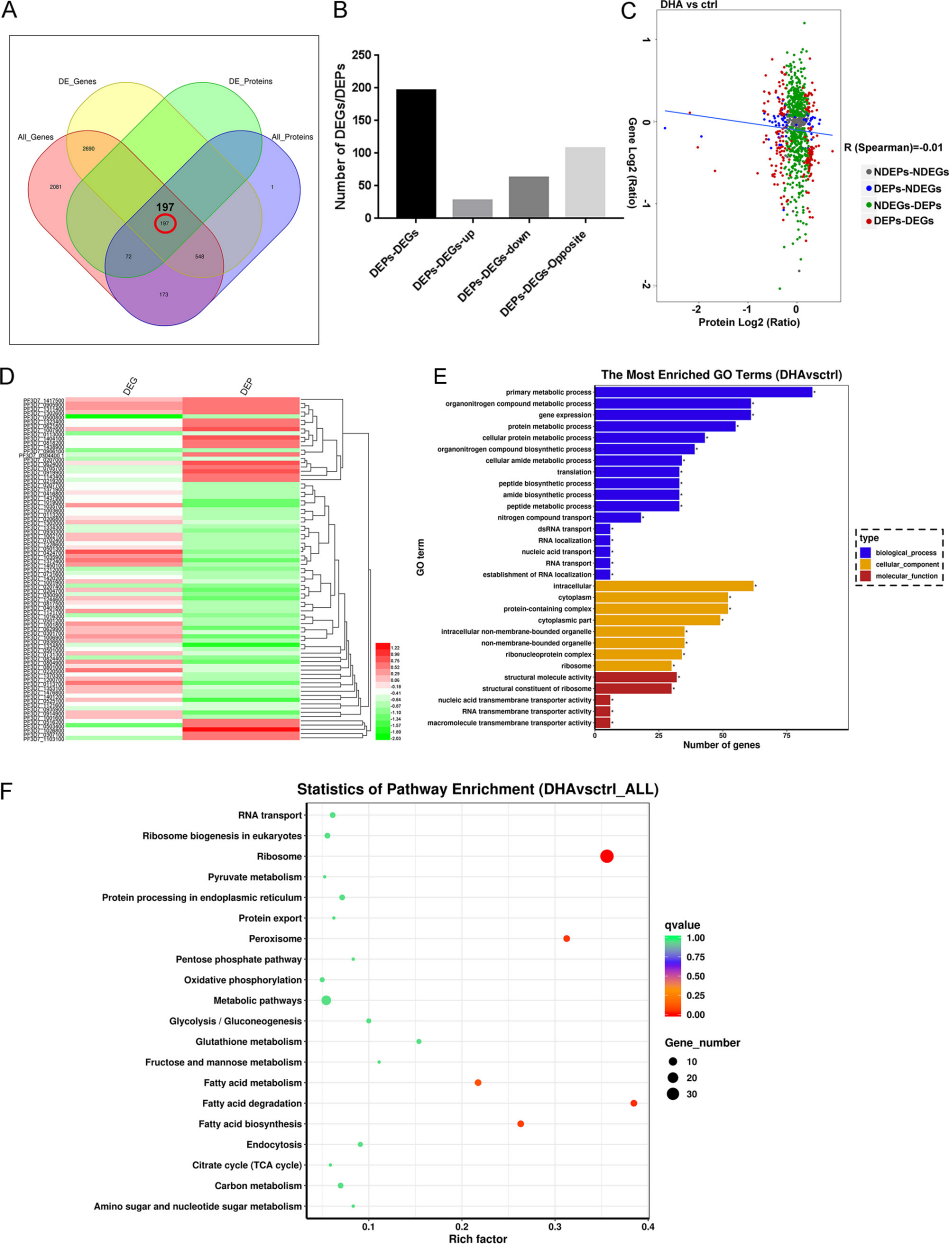
Correlation analysis of transcriptomics and proteomics. (A) Venn diagram of the numbers of all identified genes, differentially expressed genes (DEGs), all identified proteins, and differentially expressed proteins (DEPs). (B) Number of up- and downregulated genes having changes in their protein levels. (C) Correlations between protein and mRNA expression. The *x* axis represents the protein expression level, and the *y* axis represents the gene expression level. The red dots indicate DEPs and DEGs, and all data were log_2_‐transformed. The blue line here indicates linear correlation of all the proteins and genes. (D) Proteins were further subjected to unsupervised hierarchical clustering, which revealed two clusters of coregulated DEGs/DEPs. The hierarchal clustering of the log_2_ fold change (FC) (DHA/control) of transcriptomic and proteomic studies. (E) The most enriched Gene Ontology (GO) terms (*P ≤ *0.05) of DEGs/DEPs in Plasmodium falciparum 3D7. Blue, orange, and red represent GO terms belonging to biological processes, cellular components, and molecular functions, respectively. (F) Scatterplot of enriched Kyoto Encyclopedia of Genes and Genomes (KEGG) pathways. The *x* axis represents the enriched KEGG pathways, and the *y* axis represents the rich factor (ratio of the number of DEGs/DEPs enriched in a certain KEGG pathway to the number of annotated genes) of each KEGG pathway. The size of the dots indicates the number of DEGs enriched in a certain pathway, and the color of the dots corresponds to the range of the *q* value (adjusted *P* value).

The correlation of abundance changes from transcript to protein in DHA and control groups was evaluated, and the expression correlation is shown in Fig. 4. The numbers of proteins and genes correlated are described in Fig. 4A and B: 197 proteins were overlapped between DEGs and DEPs, 27 proteins were upregulated together, most metabolism proteins were found (such as deoxyhypusine hydroxylase), and 62 proteins were downregulated together, including MSRP6, GLYCOPHORIN BINDING PROTEIN, SMALL GTP BINDING PROTEIN SAR 1, and MEROZOITE SURFACE PROTEIN 4. In addition, Pearson correlation coefficient (*R*) was calculated using log_2_ transformation of the quantitative value ratio. Results showed that negligible correlation (*R* = –0.11) existed in abundance from mRNA to protein at the transcriptome and proteome levels (Fig. 4C and Table S9), indicating that the transcript contents are not perfect proxies for protein contents. The hierarchal clustering of the log_2_ FC (DHA/control) in transcriptomic and proteomic studies is shown in Fig. 4D. Results suggested that EMP1-TRAFFICKING PROTEIN, pfEMP1, and SMALL EXPORTED MEMBRANE PROTEIN 1 are downregulated in the proteome but upregulated in the transcriptome, indicating that these protein profiles might be controlled at a posttranscriptional level and changes in mRNA expression provide only limited insight into changes in protein expression.

Analysis of DEGs and DEPs with the same or opposite changes facilitates their mutual affirmation and explanation of gene/protein expression regulation. Therefore, 197 correlations were analyzed for functional classification. The GO terms in DEGs/DEPs “primary metabolic process,” “gene expression,” and “nitrogen compound transport” were significantly enriched in the BP category, whereas the term “protein-containing complex” was in the CC category and the term “structural molecule activity” was in the MF category, which are possibly related to our study (Fig. 4E). KEGG enrichment analysis showed that the relevant cluster of the KEGG pathway annotation was “metabolic pathways” and “protein export” (Fig. 4F).

### qRT-PCR analysis of *var* genes after DHA treatment.

**FIG 5:**
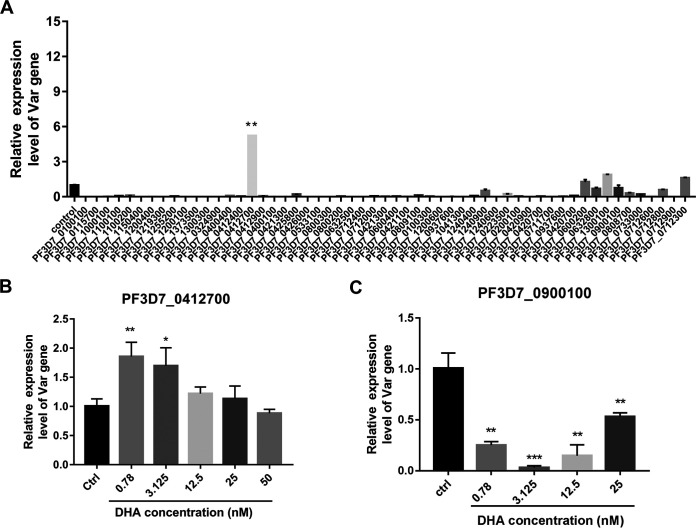
Validation of RNA-seq results. (A) 55 *var* genes were analyzed by real-time PCR. (B) mRNA extracted from Plasmodium falciparum 3D7 treated with or without DHA (0.78, 3.125, 12.5, 25, and 50 nM) for 4 h was subjected to quantitative reverse transcription PCR (qRT-PCR) to detect *PF3D7_0412700*. (C) mRNA extracted from P. falciparum 3D7 treated with or without DHA (0.78, 3.125, 12.5, and 25 nM) for 4 h was subjected to qRT-PCR to detect *PF3D7_0900100*. Data are represented as the mean ± standard deviation (SD). Student’s *t* test was performed to compare the two groups. *, *P* < 0.05, **, *P* < 0.01, and ***, *P* < 0.001 compared with the control group.

DHA treatment decreased the knobs on the external surface of iRBCs (Fig. 1A), and knobs are considered a scaffold for the presentation of the major virulence antigen pfEMP1 encoded by *var* (PMID: 31071194). Therefore, we validated the mRNA levels of 55 *var* genes of various pfEMP1 versions involved in knob formation and cytoadherence by qRT-PCR (Fig. 5). A list of primers used here is shown in Table S10. Compared with that in the dimethyl sulfoxide (DMSO) control, *PF3D7_0412700* was significantly upregulated in P. falciparum 3D7 after DHA treatment, while other *var* genes such as *PF3D7_1300100* and *PF3D7_0712300* were slightly upregulated, with most *var* genes, such as *PF3D7_0900100*, being downregulated (Fig. 5A). The changes in gene expression were consistent with the results obtained from transcriptome sequencing (RNA-seq) analysis. The validation demonstrated good reliability of RNA-seq analysis. In addition, we also detected *PF3D7_0412700* and *PF3D7_0900100* in ring-stage P. falciparum 3D7 with different doses of DHA. The mRNA expression of *PF3D7_0412700* significantly increased after 0.78 and 3.125 nM DHA treatment (Fig. 5B), while that of *PF3D7_0900100* significantly decreased after 0.78, 3.125, 12.5, and 25 nM DHA treatment (Fig. 5C).

### Verification of pfEMP1 after DHA treatment.

**FIG 6:**
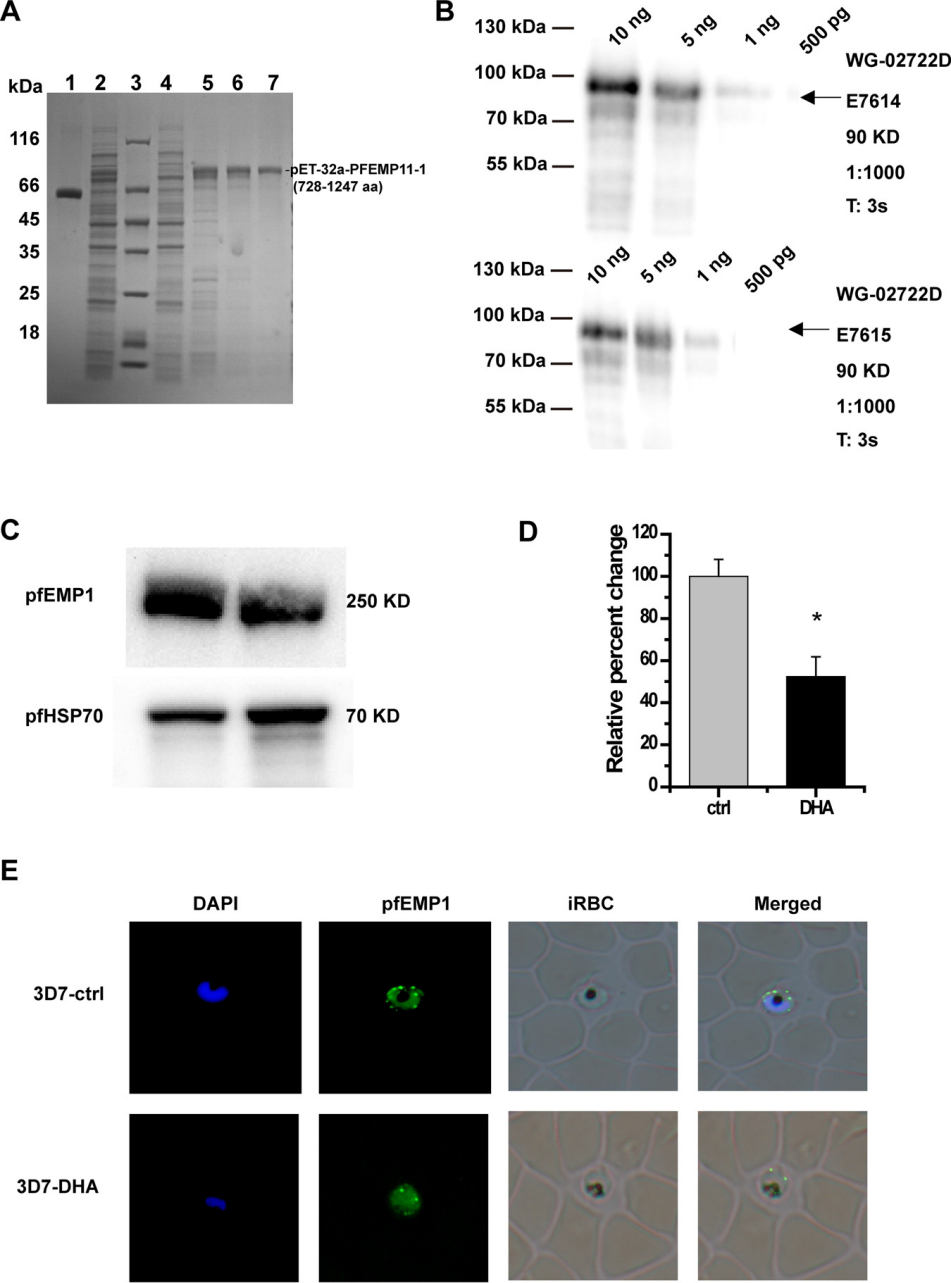
Verification of Plasmodium falciparum erythrocyte membrane protein-1 (pfEMP1) after dihydroartemisinin (DHA) treatment. (A) pfEMP1 expression and purification. Coomassie-stained sodium dodecyl sulfate-polyacrylamide gel electrophoresis (SDS–PAGE): lane 1, 0.4 mg/mL (bovine serum albumin [BSA]); lane 2, supernatant, before elution with imidazole; lane 3: marker, molecular masses in kilodaltons given to the left; lane 4: flowthrough, soluble fraction, unbound proteins; lanes 5 to 7: elute. Bound protein elution with 250, 250, and 500 mM imidazole, respectively. (B) Western blotting of antibody characterization. The eluate antigen was separated in SDS-PAGE and immunostained with E7614 and E7615 polyclonal antibodies; antigen amounts: 10 ng, 5 ng, 1 ng, and 500 pg. (C) pfEMP1 expression analysis after DHA treatment using affinity-purified antibodies by Western blotting. (D) Relative quantification of Western blot bands in panel C by densitometric analysis. (*, *P < *0.05 compared with the control group). (E) pfEMP1 expression analysis after DHA treatment using affinity-purified antibodies by immunofluorescence assay (IFA) analysis.

As shown by proteomic functional analysis, pfEMP1 was one of the hub proteins in DHA mode of action. We detected pfEMP1 using Western blotting and immunofluorescence assay (IFA) after DHA treatment. Since anti-pfEMP1 is not commercially available, we first prepared pfEMP1 polyclonal antibody using the *var* synthesis template (728 to 1,247 aa, 1,560 bp, constructed on pET-32a). After prokaryotic expression, immunization in rabbits, we finally obtain affinity-purified antibodies that recognized a single band of ∼80 kDa in Western blots (Fig. 6A and B) in extracts. Then, we detected pfEMP1 expression after DHA treatment using Western blotting and IFA. The expression of pfEMP1 on the external iRBC membrane was significantly downregulated after DHA treatment (Fig. 6C and D). IFA of pfEMP1 illustrated the weak fluorescence signal distribution in the DHA group compared with the strong fluorescence signal in DMSO controls (Fig. 6E), which is consistent with the Western blotting results.

### pfEMP1 involved in DHA mode of action for treating P. falciparum 3D7.

**FIG 7:**
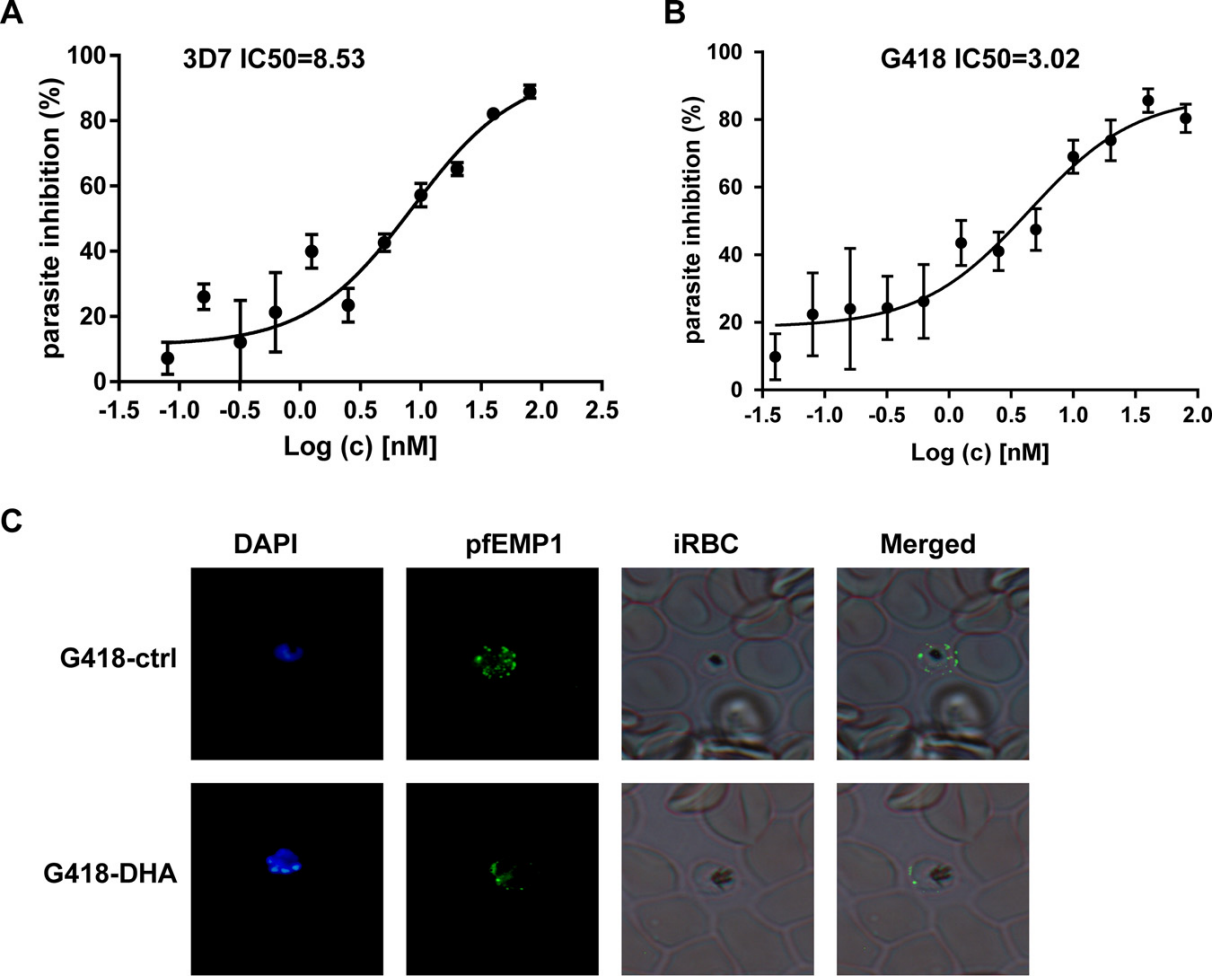
The pharmacological effect of Plasmodium falciparum erythrocyte membrane protein-1 (pfEMP1). (A, B) Median inhibitory concentration (IC_50_) of control parasites and pfEMP1-knockout parasites. (C) The validation of intracellular localization and expression of pfEMP1 after dihydroartemisinin (DHA) treatment by immunofluorescence assay (IFA).

Among both transcriptomic and proteomic validation, pfEMP1 was downregulated with 10 nM DHA treatment. In light of this evidence, we extended our study to evaluate whether the inhibition effects of DHA are retained when *var* expression is inhibited *in vivo*. We calculated the 50% infective concentration (IC_50_) of DHA in a P. falciparum line with the complete *var* repertoire silencing on the *pfrecq1* knockout (24) (provided by Jiang). After 72 h, *var*-knockout parasites showed lower IC_50_ (3.02) in the DHA group than in the control group (8.53) (Fig. 7A and B). Moreover, we used IFA to verify the intracellular localization and expression of pfEMP1 after DHA treatment. pfEMP1 expression in the iRBC membrane decreased compared to the high pfEMP1 expression in parasite cells (Fig. 7C). These results showed that it is desirable that pfEMP1 be involved in DHA mode of action.

## DISCUSSION

To develop new drugs, it is necessary to understand their mechanisms of action. In this study, we performed transcriptome sequencing analysis and iRBC membrane proteomic analysis, as well as their correlation analysis, after DHA treatment in order to highlight the possible molecular mechanism of action of artemisinin to determine the biochemical processes affected by DHA. Compared with the control group, DHA treatment affected 3,435 DEGs, of which 1,758 were downregulated and 1,677 were upregulated. These up- or downregulated DEGs may contribute to improving the antimalarial effect of DHA. The potential functions of these DEGs suggest that DHA treatment strongly and persistently affects the iRBC membrane structure, as demonstrated by the upregulated genes initiating a stress response in P. falciparum and the downregulated genes being related to the interaction with the iRBC membrane. The iRBC membrane is important in many biological processes of the parasite, such as critical cell-cell interactions, including binding to vascular endothelial cells (cytoadherence), binding to uninfected cells (rosetting), or interacting with macrophages and other leucocytes (25). In this study, most downregulated DEGs, which encode proteins exported to the iRBC membrane, such as *rif* and *stevor* (26), *pfmc-2tm* (27), and *msrp6* (28, 29), were considered promoters for triggering cytoadherence and rosetting, potentially obstructing blood flow. These findings show that DHA might lead to iRBC membrane variation, which is in agreement with Wei and Sadrzadeh, who reported that artemisinin can cause membrane damage (30). Chloroquine inhibits the aggravation of RBC deformability and the iRBC cell membrane undergoes irreversible changes (23). Therefore, DHA may change some iRBC membrane proteins but might not change the iRBC membrane structure. Further research is needed to provide evidence of the function of downregulated DEGs related to the iRBC membrane in the antimalarial effect of DHA.

Simultaneously, among the significant DEPs, 40S RIBOSOMAL PROTEIN S2 was upregulated and MSRP6 was downregulated the most. The 40S RIBOSOMAL PROTEIN S2 is a component of the 40S subunit and catalyzes protein synthesis, and it may be involved in the antimalarial action of DHA, as previous studies have suggested that many drugs can interfere with ribosome, DNA, and/or RNA metabolism and inhibit protein synthesis (31–33). MSRP6 was also downregulated, and most downregulated proteins, such as CLAG3, pfEMP1, and pfSBP1, are involved in biological processes of cell adhesion. To note, correlation analysis of DEGs and DEPs indicated that the transcript contents are not perfect proxies for protein contents. Some proteins, including pfEMP1, are downregulated in the membrane proteome but upregulated in the transcriptome, which means that these protein profiles might be controlled at a posttranscriptional level and changes in the mRNA expression provide only limited insight into changes in protein expression.

This comprehensive analysis shows that pfEMP1 expression decreases on the external surface of iRBCs. pfEMP1 is a virulence factor on the surface of iRBC and adheres to CD36 and ICAM-1 receptors on the surface of capillary endothelial cells or adheres to receptors on the surface of surrounding RBCs to form rosetting (34). This can slow the blood circulation and eventually cause blockage and have fatal consequences (35). We validated the mRNA levels of 55 *var* variants by qRT-PCR. The changes in most of these genes’ expression were consistent with RNA-seq results. Notably, alterations in the amount of *PF3D7_0412700* and *PF3D7_0900100* isoforms reduced in a DHA dose-dependent manner. The transcription profile of *PF3D7_0412700* was activated in the early stage and inactivated in the terminal stage in the DHA group, indicating that intrinsic *var* transcription is not all dependent on the genetic background, as described by Enderes et al. (36). In addition, we found that pfEMP1 expression was also significantly downregulated after DHA treatment. Most importantly, when pfEMP1 was knocked down, the IC_50_ of DHA reduced, indicating that pfEMP1 might be involved in DHA mode of action. These data confirmed that the *var* gene encoding pfEMP1 is one of the hub genes in DHA mode of action. Targeting pfEMP1 or factors regulating *var* variants is expected to be applied as potential antimalarial therapy and as adjuvant therapy in ACTs. The observed changes in the transcriptome and the membrane proteome strongly support this notion, and recent studies have also suggested exploiting chromatin and/or epigenetic regulators as drug targets in malarial diseases (37–39), providing evidence for our hypothesis of targeting pfEMP1 or factors regulating *var* variants in new antimalarial drug development.

However, whether the relationship of the IC_50_ for DHA of the infected cells with and without expression of *var* genes can be consistent with the *in vivo* actual exposure is not clear. To complement the study of the IC_50_/50% effective concentration (EC_50_) for DHA of the infected cells with and without *var* gene *in vivo*, further humanized mouse models in which a *Plasmodium* infection closely reproduces the stages of a parasite infection in humans, especially for *var* gene expression, should be constructed. Additionally, there are also many reasons why the results of *in vitro* drug IC_50_ test are not consistent with the *in vivo* clinical efficacy. First, the *in vitro* drug IC_50_ test measures the sensitivity of a single drug, while the clinical drug is a combination drug. Second, because the mechanism of action of the drug in the body is more complex than that *in vitro*, the *in vitro* single drug sensitivity test cannot reflect the synergy or addition of the drugs in the combined chemotherapy. Third, interactions of parasite-host and antimalarial drug-host are vital to the efficacy of the drugs during the *in vivo* study (40–43).

This is the first report on the human iRBC membrane proteome interaction with artemisinin. Identification of 133 membrane proteins from various subcellular locations with different functions, especially pfEMP1, would help explore the mechanism of action of artemisinin. Currently, with the reducing effectiveness of traditional antimalarial drugs, improving the therapeutic effect of ATCs is a topic of concern for scholars worldwide. Therefore, as artemisinin resistance is being increasingly reported, there is an urgent need to determine the antimalarial mechanism of artemisinin at the molecular level and provide theoretical guidance for the development of new artemisinin-based antimalarial drugs to control malaria. The comprehensive transcriptomic and proteomic analysis reported here reveals that pfEMP1 could be a promising therapeutic target and is expected to have superior performance in improving the antimalarial effects of ACTs and reducing the resistance to artemisinin. However, the problem of how DHA inhibits the cytoadherence mediated by pfEMP1 is not completely explained in this research. Further studies specifically devoted to solving the problem are needed using the *in vivo* model, such as “high-binding parasites” cytoadhesive model reported by Rowe et al. (44).

## MATERIALS AND METHODS

### Parasites and parasite culture.

P. falciparum 3D7 samples (obtained from Malaria Research and Reference Reagent Resource Center [MR4] American Type Culture Collection [ATCC]) were cultured with human erythrocytes (3% to 4% hematocrit) in RPMI medium (Sigma-Aldrich) supplemented with 0.5% Albumax II (Invitrogen) to avoid possible variations due to the use of different human serum batches and incubated at 37°C in an atmosphere of 92% N_2_, 3% O_2_, and 5% CO_2_ using standard methods. Parasite growth was monitored by counting infected erythrocytes in Giemsa-stained thin blood smears using light microscopy. Parasite growth was synchronized using 5% sorbitol and a magnetic-activated cell sorting (MACS) column.

### Morphological change in parasite cells following exposure to dihydroartemisinin.

Blood smears of 3D7 parasites were prepared at 4 and 40 h and stained with Giemsa (Biotechnical Thai, Bangkok, Thailand) to ensure the tightly synchronized parasite stages before dihydroartemisinin (DHA) treatment. Tightly synchronized ring-stage P. falciparum 3D7 cultures were exposed to DHA at the median inhibitory concentration (IC_50_, 10 nM) for 24 h until the parasites were grown to late-trophozoite stage. Then, parasite cell morphology was observed under a light microscope (×100; Olympus, Tokyo, Japan). The scanning electron microscope (SEM) was used on trophozoite-stage iRBCs obtained, as described before (45). Briefly, iRBCs were fixed in glutaraldehyde (2% in phosphate-buffered saline [PBS]) for 30 min at room temperature, washed with PBS, transferred to slides, washed again, and dehydrated using a classical protocol (45). Then, the iRBCs were dried using a critical point dryer (Balzers, Liechtenstein), attached to metallic stubs, and coated with gold for 30 s. Finally, they were observed under a Hitachi SU8100 scanning electron microscope (Hitachi, Chiba, Japan).

### RNA and library preparation for transcriptome sequencing.

Tightly synchronized ring-stage P. falciparum 3D7 cultures were exposed to DHA at the median inhibitory concentration (IC_50_, 10 nM) for 24 h until the parasites were grown to late-trophozoite stage. Total RNA was extracted from the trophozoite stage of the parasite using a total RNA extraction kit (Qiagen, Valencia, CA, USA) according to the manufacturer’s instructions. The RNA quantity and integrity were checked using a NanoDrop 2000 spectrophotometer and bioanalyzer 2100 (Agilent Technologies, Santa Clara, CA, USA). A complementary DNA (cDNA) library was constructed using a TruSeq RNA sample preparation kit v2 (Illumina, San Diego, CA, USA). Finally, sequencing libraries were constructed using a NEBNext UltraTM RNA library prep kit for Illumina (NEB, USA) according to the manufacturer’s instructions.

### RNA sequencing and differential expression gene analysis.

The libraries constructed were sequenced using an Illumina HiSeq 4000 instrument (Illumina), generating paired-end reads of 150 bp. The raw sequencing data were aligned to the P. falciparum preference genome. Raw sequencing reads in fastq format in each sample were trimmed, and clean reads were obtained by removing from the raw data reads containing adapter sequences, reads containing ploy-N, and low-quality reads. In addition, Q20, Q30, GC-content, and sequence duplication of the clean data were calculated. The clean reads were annotated against the UniProt and National Center for Biotechnology Information (NCBI) nonredundant (nr) database using BLASTX alignment with an *E* value cutoff of <1e−5. Only reads with a perfect match or one mismatch were further analyzed and annotated based on the P. falciparum genome. Tophat2 tools were used to map with the reference genome. The expression level of each gene was calculated using the fragments per kilobase of transcript per million mapped reads (FPKM). The DEGseq program and R package were used to identify differential expression analysis of control parasites and DHA-treated parasites. The *P* value was adjusted using the *q* value (46), and the criteria were a fold change (FC) of >2 and a false-discovery rate (FDR) cutoff of 5%. We used a *q* value of <0.005 and |log_2_ FC| of ≥1 for the detection of DEGs. For unigenes that were considered having differential expression, GO functional enrichment was carried out at a *P* value of <0.05. Pathway enrichment analyses (*q *< 0.05) against these DEGs was conducted using the Database for Annotation, Visualization and Integrated Discovery (DAVID) (https://david.ncifcrf.gov/), metascape (http://metascape.org/) (47), and the Kyoto Encyclopedia of Genes and Genomes (KEGG) to test the statistical enrichment of DEGs in pathway enrichment.

### Extraction of membrane fractions of iRBCs.

The synchronized trophozoite-stage 3D7 cultures were incubated with 500 nM DHA for 4 h. After that, 3D7 iRBCs, with or without DHA treatment, were MACS enriched and then were subjected to hypotonic lysis in order to prepare membrane fractions, which were treated with different extraction buffers (salt, carbonate, or 1% Triton X-100 extraction buffers, including a protease inhibitor mix) for 30 min on ice at a concentration of 1 × 10^6^ immunoelectrophoresis (IE) equivalents/μL. The membrane fractions were pelleted by centrifugation at 20,000 × *g* for 1 h at 4°C. Alternatively, they were treated with 8 M urea in 10 mM Tris at pH 8 and 1 mM ethylenediaminetetraacetic acid (EDTA) in the presence of protease inhibitors. The samples were left for 1 h at room temperature with occasional mixing at a concentration of 1 × 10^6^ iRBC equivalents/μL. Urea-soluble and urea-insoluble proteins were separated by centrifugation at 20,000 × *g* for 30 min at 4°C.

### Trypsin digestion and TMT labeling.

Protein was digested as described by Wiśniewski et al. (48). Briefly, 100 μg of protein was used for protein digestion in each sample solution with 2.5 μg of trypsin overnight at 37°C. After digestion with trypsin, sample peptides were concentrated by vacuum centrifugation using a SpeedVac system (RVC 2 to 18; Marin Christ, Osterod, Germany). Each 25- to 100-μg sample peptide was labeled with TMT label reagent for 1 h at room temperature and quenched with hydroxylamine for 15 min according to the manual of TMT mass tagging kits and reagents (Thermo Fisher Scientific, USA). Finally, the samples were combined and stored at –80°C.

### NanoLC-MS/MS analysis.

Nano-scale liquid chromatography-tandem mass spectrometry (nanoLC-MS/MS) was performed on each sample using U3000 Nano (Thermo Fisher Scientific) coupled with a Q-Exactive mass spectrometer (Thermo Fisher Scientific) via a nano-electrospray source. Reverse-phase chromatography and a trap column packed with 2-cm-long, 75-μm-inner-diameter fused silica trap column containing 3.0 μm of aqua C_18_ beads (Thermo Fisher Scientific) were used for peptide enrichment. The trap column was used for peptide loading at a flow rate of 5 μL/min, and then a fused silica analytical column (75 μm inner diameter, 25 cm length, filled with 2.0 μm aqua C18 beads; Thermo Fisher Scientific) was applied for eluting peptides at a flow rate of 300 nL/min. The eluted peptides were directly injected into the mass spectrometer via the nano-electrospray source. Ion signals were collected data dependently with the following parameters: scan range *m/z* 350 to 1,600, full scan resolution 70,000, precursor ions fragmented by high-energy collision-induced dissociation mode with MS/MS scan resolution of 3,500, isolation window 2 *m/z*, normalized collision energy of 29, and loop count 20. Dynamic exclusion was applied (charge exclusion: unassigned 1 ≥ 6; exclude isotopes: on; dynamic exclusion: 10 s; peptide match: preferred). The MS/MS file was collected and saved in raw data form using Xcalibur software version 2.2 (Thermo Fisher Scientific).

### Protein identification.

Raw MS data files were analyzed using Proteome Discoverer 2.3 (Thermo Fisher Scientific). The target sequence data set was created by downloading protein sequences of P. falciparum from the NCBI database. The following search parameters were used: trypsin specificity, up to two missed cleavages and three maximum-allowed variable posttranslational modification per peptide, and oxidation and carbamidomethyl as a variable and a fixed modification, respectively. Fragment ion tolerance was set at 0.02 Da, with precursor and mass tolerance at 15 ppm. The FDR was controlled at the protein and/or peptide level using a fusion-decoy database with a threshold of ≤1.0%. Protein identification was considered confident when at least two unique peptides with two or more spectra were recognized.

### Quantitation of protein content.

Raw MS data were processed using Proteome Discoverer 2.3 software (Thermo Fisher Scientific) to quantify protein abundance. Feature detection was performed using the expectation-maximization algorithm, and alignment of the same peptides from samples was performed using an algorithm of high-performance retention time. Next, protein levels of DHA-treated parasites were quantified, and the sum total of the three most abundant ion peak intensities of the peptides was used for protein comparison. Statistically, proteins were considered significantly altered at *P < *0.05 and FC ≥ 1.5 as the screening criteria. The protein expression profile was determined by hierarchical clustering to establish an expressional profile of differentially expressed membrane proteins between DHA and control groups and visualized using heatmap.2 and the g plots package.

### Bioinformatics analysis of the proteome.

To enrich the KEGG pathway and biological processes, the membrane differentially expressed proteins (DEPs) were submitted to Omicsbean software (http://www.omicsbean.cn/). The top enriched GO categories were shown using a right-sided hypergeometric test, comparing the background GO annotations in the P. falciparum genome. The FDR was determined using the Bonferroni step-down test *P* value correction. To better understand the protein-protein interactions (PPIs) among the DEPs of each group, we constructed PPI networks using the STRING database (http://string-db.org) with default parameters. Proteins were then grouped on the basis of their degree, with *P < *0.05.

### Hierarchical clustering analysis.

The relevant expression of some DEGs and/DEPs was applied to conduct hierarchical clustering analysis using Cluster3.0 with the Java Treeview package. A Euclidean distance measurement algorithm focusing on similarity measures and a clustering algorithm focusing on averaged linkage were selected during hierarchical clustering. In addition to visualization by a dendrogram, a heatmap was used.

### Expression of recombinant protein.

We cloned *3D7_0412700* (728 to 1,247 amino acids [aa]) encoding pfEMP1 into pET32a and expressed it in Escherichia coli Rosetta (DE3). The *3D7_0412700* DBL domain was PCR-amplified using primers 5′-CGGATCCCTGCAACACAGTGAAAACCGCACTCGAG and 3′-CTGCGGCCGCTACATGGATCACAATAATTCTCATGTC and subcloned into the expression vector Rosetta modified to contain a His tag at the C-terminal end of the construct. The expressed protein was purified by affinity chromatography with His-Trap high-performance columns (GE Healthcare). The purity of each protein was analyzed using sodium dodecyl sulfate-polyacrylamide gel electrophoresis (SDS-PAGE). His-tags were identified by Western blotting using horseradish peroxidase (HRP)-coupled anti-His antibodies (Qiagen).

### Rabbit immunization and IgG preparation.

Rabbit antisera were produced by injection of 0.6 mg of recombinant protein in Freund’s complete adjuvant, followed by three booster injections of 0.3 mg of protein in Freund’s incomplete adjuvant at 3-week intervals. Antisera were collected 2 days after the final boosting injection. The immunization-induced antibodies against recombinant pfEMP1 proteins were measured by enzyme-linked immunosorbent assay. Immunoglobulin G (IgG) was purified by manually passing rabbit immune serum through a column packed with recombinant pfEMP1 coupled to sulfolink coupling gel according to the manufacturer’s instructions, and IgG was eluted with glycine and dialyzed and concentrated overnight in PBS with 50% glycerol. Prior to Western blotting on iRBC membrane extracts, anti-pfEMP1 antibodies were preabsorbed on RBCs and incubated overnight with a membrane with a Triton X-100-insoluble/SDS-soluble fraction of RBCs to exclude a cross-reaction with RBC proteins.

### Western blotting.

The synchronized trophozoite-stage 3D7 cultures were incubated with 500 nM DHA for 4 h. After that, 3D7 iRBCs, with or without DHA treatment, were MACS enriched and then the membrane proteins were extracted as described above. The proteins were separated by SDS-PAGE using precast polyacrylamide 4% to 12% Bis-Tris gels. The nitrocellulose membrane blotted from the precast gradient Bis-Tris gels was probed with the rabbit anti-pfEMP1 antisera and/or anti-Hsp70 antibody at 1:1,000 in overnight incubation at 4°C. The reaction was revealed using Supersignal West Pico chemiluminescent substrate (Thermo Fisher Scientific).

### qRT-PCR.

Tightly synchronized ring-stage P. falciparum 3D7 cultures were exposed to DHA at the median inhibitory concentration (IC_50_, 10 nM) for 24 h until the parasites were grown to late-trophozoite stage. Total RNA was extracted from TRIzol (Invitrogen)-preserved parasites according to the manufacturer’s instructions. Following DNase I (Sigma-Aldrich) digestion of genomic DNA, cDNA was reverse-transcribed from random hexamers using Superscript II (Invitrogen) according to the manufacturer’s instructions. qRT-PCR (real-time reverse transcription PCR) was performed on the qTOWER (Jena, Germany) in 20 μL reactions using the Quantitec SYBR green PCR master mix (Qiagen). *var* gene sequences were amplified using previously validated *var* primers (49). Additional control genes were amplified using published primers seryl–tRNA synthetase (PF07_0073). The PCR cycle was as follows: 95°C for 15 min, followed by 40 cycles at 94°C for 30 s, 55°C for 40 s, and 72°C for 50 s, with a final extension at 72°C for 40 s. Transcript abundance differences between ring and trophozoite stages were determined by comparing cycle threshold (*Ct*) values in each sample run with those of the endogenous control seryl-tRNA synthetase.

### Immunofluorescence staining assay.

The synchronized trophozoite-stage 3D7 cultures were incubated with 500 nM DHA for 4 h. Immunofluorescence assay (IFA) was carried out on P. falciparum 3D7, as described previously (50, 51). Thin smears of iRBCs were made on slides and fixed with a methanol/acetone mixture. The slides were blocked in blocking buffer (10% bovine serum albumin [BSA] in 1× PBS) for 2 h at 37°C. Next, the slides were incubated with a primary antibody diluted in blocking buffer (rabbit anti-pfEMP1, 1:100) for 2 h at room temperature, then washed with PBS buffer for 30 min, and incubated with a secondary antibody conjugated to a fluorescent dye for 1 h. 4′,6-Diamidino-2-phenylindole (DAPI) was used for nuclear staining for 10 min at 37°C. The slides were washed twice with PBS (Tween 0.1%) and mounted with a cover slip. Staining was observed under a fluorescence microscope.

### Parasite growth inhibition assay for IC_50_ determination.

A 3-day SYBR green I inhibition IC_50_ analysis was performed, as described previously (52). Briefly, ring-stage parasites highly synchronized by 5% sorbitol were incubated in a 96-well plate with DHA in a 200 μL system. DHA was added at an original concentration of 80 nM with a 2-fold gradient dilution for 11 test points. The parasites were cultured for 3 days before staining with SYBR green I. The IC_50_ value and a dose-response curve were determined using GraphPad Prism.

### Data availability.

Transcriptomics data for Plasmodium 3D7 for the control and DHA treatment have been submitted to the Sequence Read Archive (SRA) with accession code PRJNA756448. Membrane proteomics data for Plasmodium 3D7 for the control and DHA treatment have been deposited at the ProteomeX change via the PRIDE partner repository with the data set identifier PRIDE: PXD028062.

## References

[1] World Health Organization. 2020. World malaria report 2020. World Health Organization, Geneva, Switzerland. https://who.int/publications/i/item/9789240015791.

[2] Dondorp AM, Nosten F, Yi P, Das D, Phyo AP, Tarning J, Lwin KM, Ariey F, Hanpithakpong W, Lee SJ, Ringwald P, Silamut K, Imwong M, Chotivanich K, Lim P, Herdman T, An SS, Yeung S, Singhasivanon P, Day NP, Lindegardh N, Socheat D, White NJ. 2009. Artemisinin resistance in Plasmodium falciparum malaria. N Engl J Med 361:455–467. doi:10.1056/NEJMoa0808859. 19641202 PMC3495232

[3] Ashley EA, Dhorda M, Fairhurst RM, Amaratunga C, Lim P, Suon S, Sreng S, Anderson JM, Mao S, Sam B, Sopha C, Chuor CM, Nguon C, Sovannaroth S, Pukrittayakamee S, Jittamala P, Chotivanich K, Chutasmit K, Suchatsoonthorn C, Runcharoen R, Hien TT, Thuy-Nhien NT, Thanh NV, Phu NH, Htut Y, Han KT, Aye KH, Mokuolu OA, Olaosebikan RR, Folaranmi OO, Mayxay M, Khanthavong M, Hongvanthong B, Newton PN, Onyamboko MA, Fanello CI, Tshefu AK, Mishra N, Valecha N, Phyo AP, Nosten F, Yi P, Tripura R, Borrmann S, Bashraheil M, Peshu J, Faiz MA, Ghose A, Hossain MA, Samad R, Tracking Resistance to Artemisinin Collaboration (TRAC), et al. 2014. Spread of artemisinin resistance in Plasmodium falciparum malaria. N Engl J Med 371:411–423. doi:10.1056/NEJMoa1314981. 25075834 PMC4143591

[4] Noedl H, Se Y, Schaecher K, Smith BL, Socheat D, Fukuda MM, Artemisinin Resistance in Cambodia 1 Study Consortium. 2008. Evidence of artemisinin-resistant malaria in western Cambodia. N Engl J Med 359:2619–2620. doi:10.1056/NEJMc0805011. 19064625

[5] Phyo AP, Nkhoma S, Stepniewska K, Ashley EA, Nair S, McGready R, Ler Moo C, Al-Saai S, Dondorp AM, Lwin KM, Singhasivanon P, Day NP, White NJ, Anderson TJ, Nosten F. 2012. Emergence of artemisinin-resistant malaria on the western border of Thailand: a longitudinal study. Lancet 379:1960–1966. doi:10.1016/S0140-6736(12)60484-X. 22484134 PMC3525980

[6] Fairhurst RM. 2015. Understanding artemisinin-resistant malaria: what a difference a year makes. Curr Opin Infect Dis 28:417–425. doi:10.1097/QCO.0000000000000199. 26237549 PMC4612278

[7] Lu F, Culleton R, Zhang M, Ramaprasad A, von Seidlein L, Zhou H, Zhu G, Tang J, Liu Y, Wang W, Cao Y, Xu S, Gu Y, Li J, Zhang C, Gao Q, Menard D, Pain A, Yang H, Zhang Q, Cao J. 2017. Emergence of indigenous artemisinin-resistant Plasmodium falciparum in Africa. N Engl J Med 376:991–993. doi:10.1056/NEJMc1612765. 28225668

[8] Talisuna AO, Karema C, Ogutu B, Juma E, Logedi J, Nyandigisi A, Mulenga M, Mbacham WF, Roper C, Guerin PJ, D’Alessandro U, Snow RW. 2012. Mitigating the threat of artemisinin resistance in Africa: improvement of drug-resistance surveillance and response systems. Lancet Infect Dis 12:888–896. doi:10.1016/S1473-3099(12)70241-4. 23099083 PMC3555126

[9] O’Neill PM, Posner GH. 2004. A medicinal chemistry perspective on artemisinin and related endoperoxides. J Med Chem 47:2945–2964. doi:10.1021/jm030571c. 15163175

[10] Cui L, Su XZ. 2009. Discovery, mechanisms of action and combination therapy of artemisinin. Expert Rev Anti Infect Ther 7:999–1013. doi:10.1586/eri.09.68. 19803708 PMC2778258

[11] Wang JG, Zhang CJ, Chia WN, Loh CC, Li Z, Lee YM, He Y, Yuan LX, Lim TK, Liu M, Liew CX, Lee YQ, Zhang J, Lu N, Lim CT, Hua ZC, Liu B, Shen HM, Tan KS, Lin QS. 2015. Haem-activated promiscuous targeting of artemisinin in Plasmodium falciparum. Nat Commun 6:10111. doi:10.1038/ncomms10111. 26694030 PMC4703832

[12] Stocks PA, Bray PG, Barton VE, Al-Helal M, Jones M, Araujo NC, Gibbons P, Ward SA, Hughes RH, Biagini GA, Davies J, Amewu R, Mercer AE, Ellis G, O’Neill PM. 2007. Evidence for a common non-heme chelatable-iron-dependent activation mechanism for semisynthetic and synthetic endoperoxide antimalarial drugs. Angew Chem Int Ed Engl 46:6278–6283. doi:10.1002/anie.200604697. 17640025

[13] Wang J, Huang L, Li J, Fan Q, Long Y, Li Y, Zhou B. 2010. Artemisinin directly targets malarial mitochondria through its specific mitochondrial activation. PLoS One 5:e9582. doi:10.1371/journal.pone.0009582. 20221395 PMC2833198

[14] Ferreira A, Balla J, Jeney V, Balla G, Soares MP. 2008. A central role for free heme in the pathogenesis of severe malaria: the missing link? J Mol Med 86:1097–1111. doi:10.1007/s00109-008-0368-5. 18641963

[15] Zhang J, Krugliak M, Ginsburg H. 1999. The fate of ferriprotorphyrin IX in malaria infected erythrocytes in conjunction with the mode of action of antimalarial drugs. Mol Biochem Parasitol 99:129–141. doi:10.1016/s0166-6851(99)00008-0. 10215030

[16] Abshire JR, Rowlands CJ, Ganesan SM, So PT, Niles JC. 2017. Quantification of labile heme in live malaria parasites using a genetically encoded biosensor. Proc Natl Acad Sci USA 114:E2068–E2076. doi:10.1073/pnas.1615195114. 28242687 PMC5358388

[17] Francis SE, Sullivan DJ, Jr, Goldberg DE. 1997. Hemoglobin metabolism in the malaria parasite Plasmodium falciparum. Annu Rev Microbiol 51:97–123. doi:10.1146/annurev.micro.51.1.97. 9343345

[18] Kavishe RA, Koenderink JB, Alifrangis M. 2017. Oxidative stress in malaria and artemisinin combination therapy: pros and cons. FEBS J 284:2579–2591. doi:10.1111/febs.14097. 28467668

[19] Dey S, Bindu S, Goyal M, Pal C, Alam A, Iqbal MS, Kumar R, Sarkar S, Bandyopadhyay U. 2012. Impact of intravascular hemolysis in malaria on liver dysfunction: involvement of hepatic free heme overload, NF-kappaB activation, and neutrophil infiltration. J Biol Chem 287:26630–26646. doi:10.1074/jbc.M112.341255. 22696214 PMC3411003

[20] Dalko E, Das B, Herbert F, Fesel C, Pathak S, Tripathy R, Cazenave PA, Ravindran B, Sharma S, Pied S. 2015. Multifaceted role of heme during severe Plasmodium falciparum infections in India. Infect Immun 83:3793–3799. doi:10.1128/IAI.00531-15. 26169278 PMC4567638

[21] Pamplona A, Ferreira A, Balla J, Jeney V, Balla G, Epiphanio S, Chora A, Rodrigues CD, Gregoire IP, Cunha-Rodrigues M, Portugal S, Soares MP, Mota MM. 2007. Heme oxygenase-1 and carbon monoxide suppress the pathogenesis of experimental cerebral malaria. Nat Med 13:703–710. doi:10.1038/nm1586. 17496899

[22] Zhang R, Lee WC, Malleret B, Suwanarusk R, Dao M, Chu C, Lim CT, Renia L, Nosten F, Russell B. 2014. Therapeutic disruption of Plasmodium vivax infected red cell deformability. Malaria J 13:O25. doi:10.1186/1475-2875-13-S1-O25.

[23] Byeon H, Ha YR, Lee SJ. 2015. Holographic analysis on deformation and restoration of malaria-infected red blood cells by antimalarial drug. J Biomed Opt 20:115003. doi:10.1117/1.JBO.20.11.115003. 26544670

[24] Li Z, Yin S, Sun M, Cheng X, Wei J, Gilbert N, Miao J, Cui L, Huang Z, Dai X, Jiang L. 2019. DNA helicase RecQ1 regulates mutually exclusive expression of virulence genes in Plasmodium falciparum via heterochromatin alteration. Proc Natl Acad Sci USA 116:3177–3182. doi:10.1073/pnas.1811766116. 30728298 PMC6386683

[25] Pasvol G, Clough B, Carlsson J. 1992. Malaria and the red cell membrane. Blood Rev 6:183–192. doi:10.1016/0268-960x(92)90014-h. 1486287

[26] Andersson A, Kudva R, Magoulopoulou A, Lejarre Q, Lara P, Xu P, Goel S, Pissi J, Ru X, Hessa T, Wahlgren M, von Heijne G, Nilsson I, Tellgren-Roth A. 2020. Membrane integration and topology of RIFIN and STEVOR proteins of the Plasmodium falciparum parasite. FEBS J 287:2744–2762. doi:10.1111/febs.15171. 31821735

[27] Sam-Yellowe TY, Florens L, Johnson JR, Wang T, Drazba JA, Le Roch KG, Zhou Y, Batalov S, Carucci DJ, Winzeler EA, Yates JR, 3rd. 2004. A Plasmodium gene family encoding Maurer's cleft membrane proteins: structural properties and expression profiling. Genome Res 14:1052–1059. doi:10.1101/gr.2126104. 15140830 PMC419783

[28] Heiber A, Kruse F, Pick C, Gruring C, Flemming S, Oberli A, Schoeler H, Retzlaff S, Mesen-Ramirez P, Hiss JA, Kadekoppala M, Hecht L, Holder AA, Gilberger TW, Spielmann T. 2013. Identification of new PNEPs indicates a substantial non-PEXEL exportome and underpins common features in Plasmodium falciparum protein export. PLoS Pathog 9:e1003546. doi:10.1371/journal.ppat.1003546. 23950716 PMC3738491

[29] Spielmann T, Hawthorne PL, Dixon MW, Hannemann M, Klotz K, Kemp DJ, Klonis N, Tilley L, Trenholme KR, Gardiner DL. 2006. A cluster of ring stage-specific genes linked to a locus implicated in cytoadherence in Plasmodium falciparum codes for PEXEL-negative and PEXEL-positive proteins exported into the host cell. Mol Biol Cell 17:3613–3624. doi:10.1091/mbc.e06-04-0291. 16760427 PMC1525250

[30] Wei N, Sadrzadeh SM. 1994. Enhancement of hemin-induced membrane damage by artemisinin. Biochem Pharmacol 48:737–741. doi:10.1016/0006-2952(94)90051-5. 8080446

[31] Burger K, Muhl B, Harasim T, Rohrmoser M, Malamoussi A, Orban M, Kellner M, Gruber-Eber A, Kremmer E, Holzel M, Eick D. 2010. Chemotherapeutic drugs inhibit ribosome biogenesis at various levels. J Biol Chem 285:12416–12425. doi:10.1074/jbc.M109.074211. 20159984 PMC2852979

[32] Ferreira R, Schneekloth JS, Jr, Panov KI, Hannan KM, Hannan RD. 2020. Targeting the RNA polymerase I transcription for cancer therapy comes of age. Cells 9:266. doi:10.3390/cells9020266. PMC7072222 31973211

[33] Baragana B, Hallyburton I, Lee MC, Norcross NR, Grimaldi R, Otto TD, Proto WR, Blagborough AM, Meister S, Wirjanata G, Ruecker A, Upton LM, Abraham TS, Almeida MJ, Pradhan A, Porzelle A, Luksch T, Martinez MS, Luksch T, Bolscher JM, Woodland A, Norval S, Zuccotto F, Thomas J, Simeons F, Stojanovski L, Osuna-Cabello M, Brock PM, Churcher TS, Sala KA, Zakutansky SE, Jimenez-Diaz MB, Sanz LM, Riley J, Basak R, Campbell M, Avery VM, Sauerwein RW, Dechering KJ, Noviyanti R, Campo B, Frearson JA, Angulo-Barturen I, Ferrer-Bazaga S, Gamo FJ, Wyatt PG, Leroy D, Siegl P, Delves MJ, Kyle DE, et al. 2015. A novel multiple-stage antimalarial agent that inhibits protein synthesis. Nature 522:315–320. doi:10.1038/nature14451. 26085270 PMC4700930

[34] Miller LH, Baruch DI, Marsh K, Doumbo OK. 2002. The pathogenic basis of malaria. Nature 415:673–679. doi:10.1038/415673a. 11832955

[35] Lalchhandama K. 2017. Plasmodium falciparum erythrocyte membrane protein 1. Wiki J Med 4:8. doi:10.15347/wjm/2017.004.

[36] Enderes C, Kombila D, Dal-Bianco M, Dzikowski R, Kremsner P, Frank M. 2011. Var gene promoter activation in clonal Plasmodium falciparum isolates follows a hierarchy and suggests a conserved switching program that is independent of genetic background. J Infect Dis 204:1620–1631. doi:10.1093/infdis/jir594. 21926380

[37] Duffy MF, Selvarajah SA, Josling GA, Petter M. 2014. Epigenetic regulation of the Plasmodium falciparum genome. Brief Funct Genomics 13:203–216. doi:10.1093/bfgp/elt047. 24326119

[38] Coetzee N, von Gruning H, Opperman D, van der Watt M, Reader J, Birkholtz LM. 2020. Epigenetic inhibitors target multiple stages of Plasmodium falciparum parasites. Sci Rep 10:2355. doi:10.1038/s41598-020-59298-4. 32047203 PMC7012883

[39] Huang Z, Li R, Tang T, Ling D, Wang M, Xu D, Sun M, Zheng L, Zhu F, Min H, Boonhok R, Ding Y, Wen Y, Chen Y, Li X, Chen Y, Liu T, Han J, Miao J, Fang Q, Cao Y, Tang Y, Cui J, Xu W, Cui L, Zhu J, Wong G, Li J, Jiang L. 2020. A novel multistage antiplasmodial inhibitor targeting Plasmodium falciparum histone deacetylase 1. Cell Discov 6:93. doi:10.1038/s41421-020-00215-4. 33311461 PMC7733455

[40] Chen M, Suzuki A, Borlak J, Andrade RJ, Lucena MI. 2015. Drug-induced liver injury: interactions between drug properties and host factors. J Hepatol 63:503–514. doi:10.1016/j.jhep.2015.04.016. 25912521

[41] Burgert L, Zaloumis S, Dini S, Marquart L, Cao P, Cherkaoui M, Gobeau N, McCarthy J, Simpson JA, Möhrle JJ, Penny MA. 2021. Parasite-host dynamics throughout antimalarial drug development stages complicate the translation of parasite clearance. Antimicrob Agents Chemother 65. doi:10.1128/AAC.01539-20. PMC8097426 33526486

[42] Prudencio M, Mota MM. 2013. Targeting host factors to circumvent anti-malarial drug resistance. Curr Pharm Des 19:290–299. doi:10.2174/138161213804070276. 22973886

[43] Burgert L, Rottmann M, Wittlin S, Gobeau N, Krause A, Dingemanse J, Möhrle JJ, Penny MA. 2020. Ensemble modeling highlights importance of understanding parasite-host behavior in preclinical antimalarial drug development. Sci Rep 10:4410. doi:10.1038/s41598-020-61304-8. 32157151 PMC7064600

[44] Claessens A, Rowe JA. 2012. Selection of Plasmodium falciparum parasites for cytoadhesion to human brain endothelial cells. J Vis Exp doi:10.3791/3122:e3122. PMC3369769 22230803

[45] Rug M, Prescott SW, Fernandez KM, Cooke BM, Cowman AF. 2006. The role of KAHRP domains in knob formation and cytoadherence of P falciparum-infected human erythrocytes. Blood 108:370–378. doi:10.1182/blood-2005-11-4624. 16507777 PMC1895844

[46] Storey JD, Tibshirani R. 2003. Statistical significance for genomewide studies. Proc Natl Acad Sci USA 100:9440–9445. doi:10.1073/pnas.1530509100. 12883005 PMC170937

[47] Tripathi S, Pohl MO, Zhou Y, Rodriguez-Frandsen A, Wang G, Stein DA, Moulton HM, DeJesus P, Che J, Mulder LC, Yanguez E, Andenmatten D, Pache L, Manicassamy B, Albrecht RA, Gonzalez MG, Nguyen Q, Brass A, Elledge S, White M, Shapira S, Hacohen N, Karlas A, Meyer TF, Shales M, Gatorano A, Johnson JR, Jang G, Johnson T, Verschueren E, Sanders D, Krogan N, Shaw M, Konig R, Stertz S, Garcia-Sastre A, Chanda SK. 2015. Meta- and orthogonal integration of influenza “OMICs” data defines a role for UBR4 in virus budding. Cell Host Microbe 18:723–735. doi:10.1016/j.chom.2015.11.002. 26651948 PMC4829074

[48] Wiśniewski JR, Zougman A, Nagaraj N, Mann M. 2009. Universal sample preparation method for proteome analysis. Nat Methods 6:359–362. doi:10.1038/nmeth.1322. 19377485

[49] Jiang L, Mu J, Zhang Q, Ni T, Srinivasan P, Rayavara K, Yang W, Turner L, Lavstsen T, Theander TG, Peng W, Wei G, Jing Q, Wakabayashi Y, Bansal A, Luo Y, Ribeiro JM, Scherf A, Aravind L, Zhu J, Zhao K, Miller LH. 2013. PfSETvs methylation of histone H3K36 represses virulence genes in Plasmodium falciparum. Nature 499:223–227. doi:10.1038/nature12361. 23823717 PMC3770130

[50] Ramasamy G, Gupta D, Mohmmed A, Chauhan VS. 2007. Characterization and localization of Plasmodium falciparum homolog of prokaryotic ClpQ/HslV protease. Mol Biochem Parasitol 152:139–148. doi:10.1016/j.molbiopara.2007.01.002. 17270290

[51] Mohmmed A, Kishore S, Patra KP, Dasaradhi PV, Malhotra P, Chauhan VS. 2005. Identification of karyopherin beta as an immunogenic antigen of the malaria parasite using immune mice and human sera. Parasite Immunol 27:197–203. doi:10.1111/j.1365-3024.2005.00759.x. 15987343

[52] Smilkstein M, Sriwilaijaroen N, Kelly JX, Wilairat P, Riscoe M. 2004. Simple and inexpensive fluorescence-based technique for high-throughput antimalarial drug screening. Antimicrob Agents Chemother 48:1803–1806. doi:10.1128/AAC.48.5.1803-1806.2004. 15105138 PMC400546

